# Supplementary material for: Drug repurposing for aging research using model organisms
Source: Aging Cell. 2017 Jun 16;16(5):1006–15. doi: 10.1111/acel.12626 (PMC5595691; doi:10.1111/acel.12626)
Supplement: Supplementary file 7 — Data S1 Zip‐Archive of all report cards. [file ACEL-16-1006-s007.zip › RC_CYS.pdf]

CYS

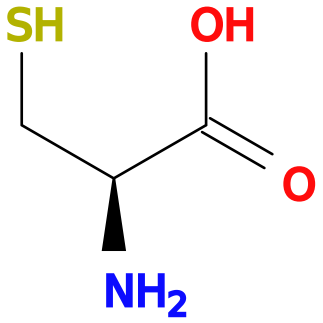

Database identifiers

ChEMBLCompound	CHEMBL863
DrugBank	DB00151
DrugBank	DB03201
CHEBI	17561
CHEBI	35235
ZINC	ZINC00895042
eMolecules	484930

Ranking

	Rank	Score
Drosophila	491/697	0.286
C. elegans	219/591	0.236

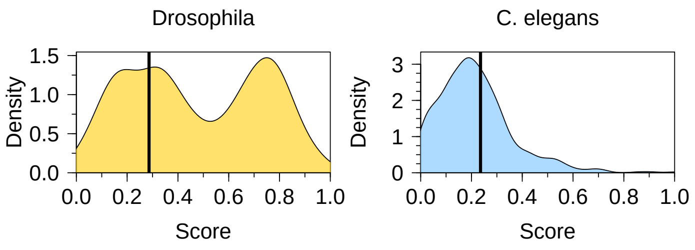

	Ageing implication	Domain conservation	Binding site conservation	Binding affinity	Bioavailability	Lipinski	Promiscuity	Purchasability	Drug approval	**Total**
Drosophila	1.0	0.922	0.379	0.276	(0.9)	0.0	-0.0	0.1	0.1	0.286
C. elegans	1.0	0.748	0.74	0.276	0.237	0.0	-0.0	0.1	0.1	0.236

Names

- **L-cysteine zwitterion**
- (2R)-2-ammonio-3-mercaptopropanoate
- Cysteine

Roles

ChEBI entry 35235 has no roles

Status

Approved drug (according to ChEMBL)	Yes
Number of Rule of 5 violations	0
Binding affinity to original target in log units (RF-Score prediction)	4.03
Burns *C. elegans* bioavailability prediction	-2.81

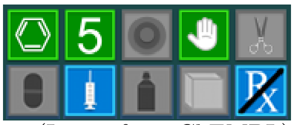

(Image from ChEMBL)

Compound Target Characteristics

Superoxide dismutase [Cu-Zn]

Best gene implication in ageing for this target family came from gene P61851 annotated in UniProt release 2014_02. Annotation GO 8340 (determination of adult lifespan) was Inferred from Mutant Phenotype

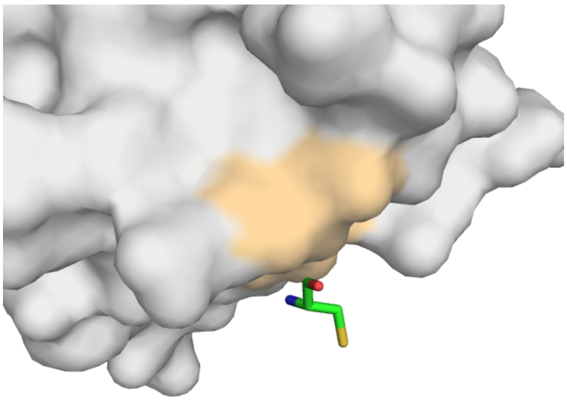

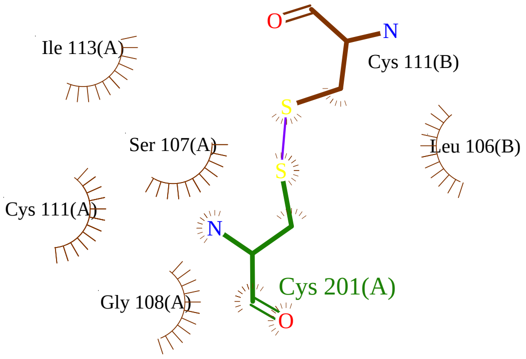

protein	amino acids contacts (binding site)			
PDB:4ff9:chainA:P00441	S	G	C	I
tr:H7BYH4:H7BYH4_HUMAN	S	G	C	I
sp:P00441:SODC_HUMAN	S	G	C	I
sp:P07632:SODC_RAT	S	G	S	I
tr:Q6LDS4:Q6LDS4_RAT	S	G	S	I
sp:P08228:SODC_MOUSE	S	G	S	I
sp:P61851:SODC_DROME	F	G	S	I
tr:G5ECR5:G5ECR5_CAEEL	S	G	S	I
sp:P34461:SODE_CAEEL	S	G	S	I
sp:P00445:SODC_YEAST	I	G	S	V

	whole protein		domain-based		contact-based	
protein	ident	simil	ident	simil	ident	simil
PDB:4ff9:chainA:P00441	1.0	1.0	1.0	1.0	1.0	1.0
tr:H7BYH4:H7BYH4_HUMAN	0.87	0.87	0.87	0.87	1.0	1.0
sp:P00441:SODC_HUMAN	1.0	1.0	1.0	1.0	1.0	1.0
sp:P07632:SODC_RAT	0.83	0.94	0.83	0.94	0.75	0.74
tr:Q6LDS4:Q6LDS4_RAT	0.82	0.93	0.82	0.93	0.75	0.74
sp:P08228:SODC_MOUSE	0.84	0.94	0.83	0.94	0.75	0.74
sp:P61851:SODC_DROME	0.61	0.84	0.6	0.85	0.5	0.38
tr:G5ECR5:G5ECR5_CAEEL	0.35	0.57	0.44	0.71	0.75	0.74
sp:P34461:SODE_CAEEL	0.35	0.57	0.44	0.71	0.75	0.74
sp:P00445:SODC_YEAST	0.54	0.83	0.55	0.84	0.25	0.41

Sod (FBgn0003462) associated phenotypes

calorie restriction conditional, chemical conditional, chemical sensitive, heat sensitive, increased cell number, locomotor behavior defective, long lived, male limited, oxidative stress response defective, partially, partially lethal - majority die, progressive, radiation sensitive, reduced, short lived

(Information from FlyBase)

Sod (UniProt:P61851) annotation
Function: Destroys radicals which are normally produced within the cells and which are toxic to biological systems.
Cofactor:Cu cationNote=Binds 1 copper ion per subunit.;
Cofactor: Zn(2+)Note=Binds 1 zinc ion per subunit.;
Subunit: Homodimer.
Subcellular location: Cytoplasm.

(Information from UniProt)

sod-4 (UniProt:G5ECR5) annotation
Function: Destroys radicals which are normally produced within the cells and which are toxic to biological systems. (RuleBase:RU000393).
Cofactor: Cu cation Evidence=(RuleBase:RU000393); Note=Binds 1 copper ion per subunit. (RuleBase:RU000393);
Cofactor: Zn(2+) Evidence=(RuleBase:RU000393); Note=Binds 1 zinc ion per subunit. (RuleBase:RU000393);

(Information from UniProt)

sod-4 (UniProt:P34461) annotation
Function: Destroys radicals which are normally produced within the cells and which are toxic to biological systems.
Cofactor:Cu cationNote=Binds 1 copper ion per subunit. ;
Cofactor: Zn(2+)Note=Binds 1 zinc ion per subunit. ;
Subcellular location: Isoform 1: Secreted, extracellular space.
Subcellular location: Isoform 2: Membrane ; Peripheral membrane protein
Tissue specificity: Isoform 2 is preferentially expressed in eggs.

(Information from UniProt)
